# Markers of Treatment Response to Methotrexate in Rheumatoid Arthritis: Where Do We Stand?

**DOI:** 10.1155/2012/978396

**Published:** 2012-07-09

**Authors:** Karina I. Halilova, Elizabeth E. Brown, Sarah L. Morgan, S. Louis Bridges, Min-Ho Hwang, Donna K. Arnett, Maria I. Danila

**Affiliations:** ^1^Division of Clinical Immunology and Rheumatology, The University of Alabama at Birmingham, Birmingham, AL 35294, USA; ^2^Department of Epidemiology, The University of Alabama at Birmingham, Birmingham, AL 35294, USA

## Abstract

Methotrexate (MTX) is the most commonly used disease-modifying antirheumatic drug (DMARD) for the treatment of rheumatoid arthritis (RA). However, despite its efficacy and affordability, additional DMARDs or biologic agents are often required in order to achieve the recommended goals of low disease activity or remission. Although well tolerated by most, some patients develop important side effects such as cytopenias, gastrointestinal adverse events (stomatitis, nausea), or abnormal liver function tests, which may limit its use and may result in additional health care costs. Given the clinical implications of widespread use of MTX in RA, various studies have evaluated the role of potential biomarkers in predicting treatment effectiveness of MTX. These biomarkers include RBC MTX polyglutamate (PG) levels; genetic variation in genes from relevant biological and metabolic pathways; gene expression profiles; serum proteins. This paper provides an update on the current data regarding biomarkers of treatment response to MTX.

## 1. Introduction

MTX is known to be a potent anti-inflammatory and immunosuppressant agent that acts by decreasing cell proliferation, increasing adenosine release, and inhibiting enzymes of folate metabolism [1]. MTX also modifies the expression of cellular adhesion molecules, alters production of cytokines, and has effects on humoral responses, and bone formation, and deposition [2]. MTX is the anchor DMARD for the treatment of RA and other types of inflammatory arthritis (psoriatic arthritis, juvenile idiopathic arthritis, etc.) because of its efficacy in decreasing articular inflammation and preventing joint damage [3]. Since its use became wide spread in the 1980s, MTX has dramatically improved RA outcomes [4]. In spite of its affordability [5], MTX is not universally effective and in some patients is associated with clinically significant side effects such as cytopenias, liver function test abnormalities, and rarely lymphoma, and other serious conditions. The introduction of biologic DMARDs in the past 15 years has further revolutionized the treatment of RA and juvenile idiopathic arthritis (JIA), but these newer drugs are more expensive than MTX and have also potential side effects.

 Due to the complexity of RA pathogenesis and the heterogeneity of disease manifestations and severity [6], there is substantial variability in how patients respond to each DMARD, be it MTX or a biologic DMARD. For example, approximately 30–40% of patients do not have a good response to MTX despite optimal dosing regimens [7]. Notwithstanding the great deal of interest in the discovery of biomarkers of treatment response and toxicity to DMARDs in RA and other types of inflammatory arthritis, there is a paucity of reliable, clinical-grade markers of treatment response or toxicity to MTX and other DMARDs available in clinical practice [8]. Multiple factors such as RA disease duration, autoantibody [rheumatoid factor (RF) or anti cyclic citrullinated peptide antibody (ACPA)] status, or smoking status can influence treatment response to different medications in patients with RA. Using analysis of genetic variants, biochemical assays, and proteomics approaches, several promising biomarkers for toxicity and treatment response have been proposed, including red blood cell (RBC) MTX polyglutamate levels, single nucleotide polymorphisms (SNPs) and other genetic variants, and gene expression levels in peripheral blood cells, as well as serum levels of proteins such as cytokines, growth factors, and autoantibodies. This paper provides an update on the current data regarding biomarkers of treatment response to MTX.

 The ideal biomarker for treatment response and toxicity should be widely available, easily measurable, accurate, reproducible, and inexpensive. Improved understanding of biological markers of MTX treatment response and the mechanism of action of MTX may be helpful, not only in identifying RA patients who are most likely to respond to MTX, but also those who may respond unfavorably, such as those who may develop infections or other toxicities.

## 2. Clinical, Radiographic, and Biochemical Correlates of MTX Response

 Because of the relative ease of access to clinical and demographic parameters, many investigators have evaluated whether clinical factors can be used to predict the response to MTX, but studies have reported contradictory results. A recent systematic review of predictors of RA remission found that demographic and clinical characteristics of RA (such as male sex; young age; late-onset RA; low disease activity; RF status; ACPA status; nonsmoker status; short disease duration; mild functional impairment; low baseline radiographic damage) correlated with a higher rate of remission in patients with RA [10]. In a recent study of 124 Japanese RA patients treated with various DMARDs (most commonly MTX), 40% of patients developed resistance to DMARDs during the followup period of 2 years. After adjustment for age at disease onset, RF status, and prednisolone use, two factors were found to be associated with treatment resistance: HLA DRB1* 04 alleles encoding the shared epitope (OR, 2.89; 95%CI, 1.28–6.53; *P* = 0.011), and ACPA status (OR, 6.31; 95%CI, 1.23–32.34; *P* = 0.027) [11]. However, a study of 309 patients with inflammatory polyarthritis on MTX from the Norfolk Arthritis Register found that clinical and laboratory factors such as age, gender, age at disease onset, baseline RF, and CRP were poor predictors of treatment response to MTX [12].

 In a secondary analysis of participants in the Swedish Pharmacotherapy trial (SWEFOT), which included 487 RA patients with a symptom duration of less than 1-year receiving MTX monotherapy [13], a poor response to MTX correlated with longer symptom duration and younger age. In addition, current smoking status and female gender were also associated with suboptimal response to MTX [14]. In the Pediatric Rheumatology International Trials Organization (PRINTO) study of patients with polyarticular JIA taking MTX [15], participants with longer disease duration, higher disability (quantified using patient-centered disability measures), active wrist arthritis, and without antinuclear antibodies at baseline were more likely to have a suboptimal response after a 6-month course of MTX [16].

 Uncontrolled inflammation in RA leads to bone damage and the appearance of periarticular osteopenia and marginal erosions. Conventional radiographs of the hands and feet have been routinely used to assess the degree of joint damage in patients with RA and to evaluate for progression of disease, both in current clinical practice as well as in the clinical trials of new therapeutic agents. In fact, U.S. Food and Drug Administration (FDA) requires that in order to claim prevention of structural damage on their commercial label, new investigational agents proposed for the treatment of RA need to be assessed for their ability to slow radiographic progression as measured by a validated radiographic index such as the Sharp/van der Heidje score [17–19].

 Although radiographs of the hands and feet have been traditionally used to document RA severity and to assess response to conventional (MTX) and biologic DMARDs [20, 21], they are relatively insensitive to detection of early erosions. Musculoskeletal ultrasound is a dynamic study of joints with very high sensitivity for inflammation and bony erosions compared with plain radiography in RA, which cannot detect active inflammation, but rather the result of long-standing inflammation. Gray-scale and power Doppler musculoskeletal ultrasound have been shown to be useful technologies in evaluating synovitis and the MTX treatment response in RA [22], and more recently has been proposed as a secondary outcome in clinical trials [23, 24].

## 3. Cytokines

 Taking into consideration the complexity of RA manifestations and patient-to-patient variability (age, sex, and comorbidities), the prediction of treatment response in individuals to ultimately allow selection of targeted, patient-specific therapy will likely be based on novel and integrative biomarker approaches. Therefore, an area of intense investigation has been in exploring the ability of cytokines to molecularly characterize treatment response in RA. Cytokines have a pivotal role in pathogenesis of RA [6], and their cellular production and metabolism is influenced by MTX. For example, MTX was found to inhibit cytokine production in T cells, but not in monocytes [25]. In addition, MTX decreases the production of IL-1, IL-4, IL-6, IL-13, TNF-*α*, IFN-*γ*, and GM-CSF and may reduce cell adhesion and the regulation of IL-15, IL-8, CD69, CD25, and IL-17 [25–27].

 Several inflammatory cytokines have been evaluated for their potential to predict MTX treatment response, especially in early stages of the disease, prior to the development of severe joint damage. In a study of 50-consecutive RA patients, patients with good or excellent responses to MTX treatment had a significantly lower ratio of IL-1ra/IL-1beta (ratio < 100), cytokines constitutively produced by peripheral blood mononuclear cells (PBMCs) [28]. In addition, serum TNF-*α* concentrations above 20.1 pg/mL were negatively correlated to treatment response of MTX at 6 months in 42 RA patients [29], while serum IL-1*β*, IL-6, IL-8, IL-10, and IL-12 level and expression of multidrug resistance protein (encoded by *MDR1*) in PBMCs did not correlate with response to MTX [29]. Taken together, these studies suggest that cytokines are promising candidates as biomarkers of treatment response to MTX.

## 4. RBC MTX Polyglutamates

**Figure 1:**
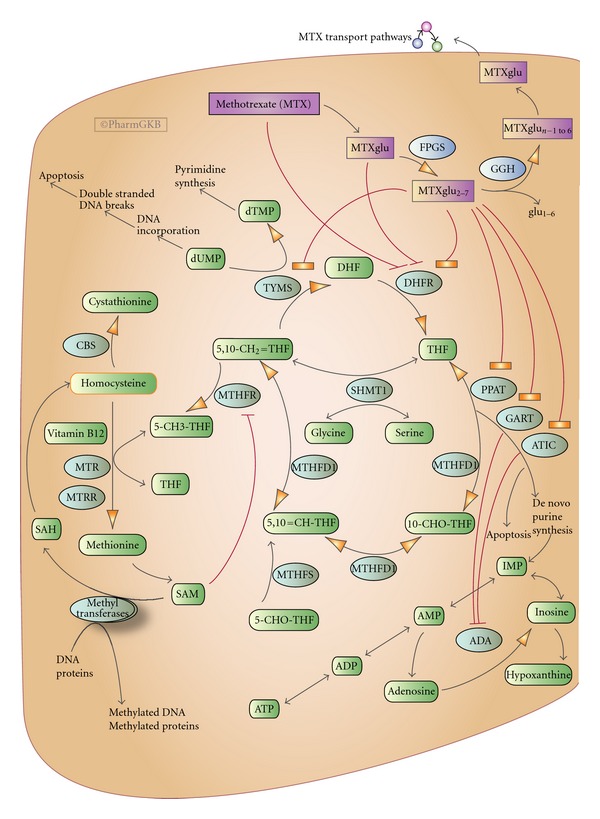
Methotrexate cellular disposition and effects from reference [9]. Copyright PharmGKBu, Used with permission from PharmGKB and Stanford University.

 RBC MTX polyglutamate (MTX PG) concentrations have been proposed as biomarkers of MTX response in patients with RA [30]. MTX enters cells through interaction with the reduced folate carrier (RFC). MTX is then subjected to polyglutamylation by the enzyme FPGS (folylpolyglutamate synthetase) (Figure 1). A variable number of glutamate residues may be added, yielding molecules of different lengths. The MTX PGs range from MTX PG 1 (MTX monoglutamate) to MTX PG 5 (pentaglutamate) [31] and can be classified as MTX PG 1-2 (short chain), MTX PG 3 (long chain), and MTX PG 4-5 (very long chain), with the MTX PG 3 being the most common intracellular form of MTX PG [32]. MTX PGs are the active MTX metabolites that produce the anti-inflammatory effects and inhibit enzymes of folate metabolism [33, 34]. MTX PG levels are influenced by age, renal function, MTX dose and route of administration and treatment duration, smoking, concurrent use of other medications such as other DMARDS, and corticosteroids and nonsteroid anti-inflammatory drugs [31, 35–37].

 A series of studies conducted by Dervieux et al. found that long-chain [30, 38, 39] and short-chain [40] RBC MTX PGs levels correlated with improved clinical outcomes as measured by 28-joint Disease Activity Score (DAS28). However, other investigators have found that total, long, and very long chain MTX PG concentrations were not associated with RA disease control in long-term MTX therapy recipients [41]. In conclusion, at the present time, more research is needed before MTXPG-level measurement is ready for routine clinic use to guide MTX dosing.

## 5. Genetic Variants

 The results of recent studies investigating genetic factors of MTX effectiveness have been conflicting likely due to lack of sufficient statistical power, various clinical and pharmacological confounders, and heterogeneity of phenotypes [42]. The methodological designs of the pharmacogenetic studies are mainly based on candidate genes/SNPs leading to limitations of the true interpretation of actual factors influencing the treatment outcomes [8]. The *HLA-DRB1* shared epitope (SE) has been shown to be associated with RA severity and disease progression [43–45], but studies on whether it is associated with treatment response to MTX and other DMARDs have yielded conflicting results. In a study of 457 RA patients, the presence of two *HLA-DRB1* alleles encoding the SE were associated with good treatment response to etanercept as compared to MTX [46]. In another study, SE-positive patients responded better to MTX, sulfasalazine, and hydroxychloroquine combination therapy compared to MTX alone, while SE-negative patients responded well irrespective of treatment [47]. In a Japanese population, carriers of SE-positive *04 alleles were more likely to develop resistance to DMARDs including MTX compared to noncarriers [11].

 Because MTX exerts its anti-inflammatory and immunosuppressant activities through inhibition of folate-dependent pathways and adenosine release, it was suggested that genetic polymorphisms in these pathways may influence response to MTX among RA patients [48]. In 2007, Wessels et al. [49] developed a clinical pharmacogenetic model based on previously published SNP associations to predict MTX efficacy based on data from 205 RA patients. In addition to baseline variables, the authors analyzed 17 genetic polymorphisms in 13 genes important for the MTX mechanism of action, including purine and pyrimidine synthesis pathways. They constructed a model that included sex, smoking status, RF presence, DAS28, and 4 polymorphisms in the *AMPD1* (adenosine monophosphate deaminase), *ATIC* (aminoimidazole carboxamide ribonucleotide transformylase), *ITPA* (inosine triphosphate pyrophosphatase), and *MTHFD1* (methylene-tetrahydrofolate reductase). They categorized patients into three groups: nonresponders, intermediate responders, and good responders to MTX therapy. Patient responses were assessed by a simple-scoring system that ranged from 0 to 11.5. Patients scoring ≤3.5 had a true positive response at 95%; patients scoring ≥6 had a true negative response at 86%.

 Genetic studies of MTX efficacy have also included children. The SNPs of the 13 genes (*ABCG2, ADORA2A, AMPD1, ATIC, DHFR, FPGS, GGH, ITPA, MTHFD1, MTHFR, SHMT1, SLC19A1, *and* TYMS* in the MTX pathway were tested for association with MTX efficacy in two populations of children with JIA (197 children from UK CHARMS [Sparks Childhood Arthritis Response to Medication Study] and 210 children from a US cohort). Of these 13 genes, one SNP within *ITPA *and two SNPs within *ATIC* had statistically significant associations with poor MTX treatment response in the UK CHARMS cohort. In the US-based cohort, a SNP in the ATIC gene (rs12995526, which has a high degree of linkage disequilibrium with rs13005416) had significant associations with poor MTX treatment response; this finding was further supported by a meta-analysis of two independent studies (*P* = 0.002) [50]. Lee et al. evaluated the association between candidate SNPs in *ATIC*, *ITPA,* and *MTHFR* with DAS28 and C-reactive protein (CRP) levels in 556 participants from the Brigham and Women's Hospital Rheumatoid Arthritis Sequential (BRASS) study. They found that *ATIC* SNP rs4673993 was associated with low DAS28 score (*P* = 0.04) in patients who were on MTX monotherapy or combination therapy. In another study of 281 North Indian RA patients, variants within genes in the purine biosynthesis pathway were evaluated, and *FPGS* rs1544105, *TYMS* rs2853539, *DHFR* rs7387, and *ADA* rs244076 were identified as potential predictors of MTX response [51, 52].

## 6. Gene Expression Patterns of Treatment Response to MTX

 Evaluation of the association between gene expression patterns and disease activity could be useful for monitoring RA disease activity and MTX treatment effectiveness in RA patients. For example, RA synovial expression of genes encoding collagenase, stromelysin, and tissue inhibitor metalloproteinase 1 (TIMP-1) was studied. After MTX treatment, collagenase, but not stromelysin or TIMP-1 gene expression, was found to be significantly decreased in the synovium from patients with RA [53]. Moreover, examination of gene-expression profile of selected RA-related genes in synovial fibroblasts revealed that MTX administration decreased the gene expression of insulin-like growth factor binding protein 3, retinoic acid induced 3, and caveolin 2 [54].

 Gene expression of IL-4 and IL-10 were significantly increased in PBMC from RA patients than in healthy controls after MTX exposure in vitro, while gene expression of IL-2 and IFN*γ* was significantly decreased [55]. Seitz et al. showed increased release of IL-1*β* in PBMC from RA patients who had good or excellent response to MTX [28]. In a recent study, Galligan et al. demonstrated that MTX therapy resulted in increased expression of 11 genes: *MCTP2, ALDH1A2, CASP1, ESR1, VAV3, MBP, TM4SF12, CPNE3, PCSK1, SLC16A4*, and *SERPINF1*, and reduced gene expression of 26 genes, including *ICAM1, RGS16*, *GATA6,* and others [56]. Furthermore, Hobl et al. showed that MTX monotherapy reduced the expression of IL-12A in PBMC from RA patients (*P* < 0.046) and that the combination of MTX and corticosteroids decreased expression of the IL18 gene [57]. In another study, there was higher expression of bone morphogenetic protein-2 (BMP-2) and ligand for herpesvirus entry mediator (LIGHT) in RA patients treated with MTX versus controls [58]. These studies are laying the foundation for the development of gene expression panels that could be used by clinicians in the hopefully not distant future. For example, gene expression profiling for response to infliximab and other biologic agents is currently underway [59].

## 7. Future Directions

 Identifying biomarkers of treatment response and toxicity to MTX and other DMARDs is critically important for advancing the field of personalized medicine in RA [60, 61]. As such, in recent years there has been a heightened interest in focusing collective research efforts on the discovery of biomarkers of disease activity and treatment response. In this regard, high quality, large prospective pharmacogenetic studies involving well-phenotyped participants are being developed in order to examine and confirm the role of various candidate genes in MTX efficacy and toxicity.

 Despite much effort, rheumatologists currently lack reliable and inexpensive clinical grade biomarkers to individualize treatment choices in managing RA. Therefore, innovative, well-powered, and collaborative translational research projects are needed to bring affordable, reliable, and clinically useful tests for individualized treatment into clinical realm. Large multicenter collaborative networks, repositories, and databases such as the Treatment Efficacy and Toxicity in Rheumatoid Arthritis Database (TETRAD) [62], and others have been established to provide the foundation for future studies of biomarkers of disease response and to provide a detailed understanding of RA phenotypes. The unique resources provided by these large-scale collaborations depend on continued collaborative efforts of academia, federal agencies, industries, and other donor organizations.

 The results of recent gene expression studies [28, 54, 56–58] demonstrating the effect of MTX on PBMC, and synovial cells will likely play a key role in the future research of biomarkers of response to DMARDs and biologic agents in RA. Stratification of RA patients using molecular phenotyping such as detailed subtyping of ACPA response [63], single-cell network profiling [64], genetic profiling [65], or other techniques may be useful in providing better targeted therapies. There is a great need for new bioinformatics approaches and better statistical methods that can integrate clinical, genetic, and other biomarker data and construct clinically useful valid algorithms to allow accurate prediction of MTX treatment response.
